# Attenuation of species abundance distributions by sampling

**DOI:** 10.1098/rsos.140219

**Published:** 2015-04-22

**Authors:** Hideyasu Shimadzu, Ross Darnell

**Affiliations:** 1 Centre for Biological Diversity and Scottish Oceans Institute, University of St Andrews, Dyers Brae House, St Andrews, Fife KY16 9TH, UK; 2 Geoscience Australia, GPO Box 378, Australian Capital Territory 2601, Australia; 3 The Commonwealth Scientific and Industrial Research Organisation, PO Box 2583, Brisbane, Queensland 4001, Australia

**Keywords:** biodiversity estimation, marine surveys, rarefaction, species presence/absence, richness, sampling

## Abstract

Quantifying biodiversity aspects such as species presence/ absence, richness and abundance is an important challenge to answer scientific and resource management questions. In practice, biodiversity can only be assessed from biological material taken by surveys, a difficult task given limited time and resources. A type of random sampling, or often called sub-sampling, is a commonly used technique to reduce the amount of time and effort for investigating large quantities of biological samples. However, it is not immediately clear how (sub-)sampling affects the estimate of biodiversity aspects from a quantitative perspective. This paper specifies the effect of (sub-)sampling as attenuation of the species abundance distribution (SAD), and articulates how the sampling bias is induced to the SAD by random sampling. The framework presented also reveals some confusion in previous theoretical studies.

## 2. Introduction

Assessing biodiversity measures in species communities, such as species presence/absence, richness and abundance, has been an important task for many aspects of ecological studies including environmental research, resource management and conservation planning. As the observations we can obtain in ecological studies are often a consequence of sampling from the population of interest, a key challenge in assessing biodiversity is dealing properly with the uncertainty induced by sampling.

Owing to limited time and resources, it is almost impossible to collect the entire species community for identification, counting and weighing, forcing us to take a part of the entire community, or *sampling*, for investigation. However, whenever a large number of specimens are caught in surveys, collecting a part of the whole catch, *sub-sampling*, is one solution that has been widely used to deal with this difficulty. The prefix ‘sub-’ implies the fact that the whole catch is already taken, as a sample from the entire species community of interest, so the part of the whole catch is yet another random sample but from the sample already caught. Although it may be slightly misleading to mix up those terms sampling and sub-sampling, from a statistical perspective which draws inferences on the population of interest, we however treat these terms as the same meaning throughout this paper. In other words, our population of interest is the situation before a sample or a sub-sample is taken in a general context. We focus on the issue of information loss by (sub-)sampling; the act of sampling nonetheless attenuates the true information of the species community as we discuss in the later sections.

An early study on the effect of sampling in ecological study can be found in Sanders [1], who has investigated the difference between his sub-sample and sample (the whole catch) of marine benthic organisms. The species richness (number of species) of the sub-sample was lower (negatively biased) than the one of the entire catch. Although this negative bias might sound contradictory, based on our knowledge from random sampling theory, this bias is largely due to the fact that ecological sampling is commonly done based on individuals (or equivalent) but not on species. Small abundance species have lower probability of being sampled than more abundant ones, which leads to a bias in species richness whenever random sampling is undertaken. Sanders' initial idea of *rarefaction* that corrects for the bias, later refined by Hurlbert [2], Simberloff [3] and Heck *et al*. [4], allows for calculation of the expected species richness when sub-samples are taken. The most important implication from the Sanders' observation was that the resulting rarefaction of richness is a consequence of imperfect observation of *individuals* by sampling.

Clearly, species richness is not the only measure affected by sampling. Species abundance distributions (SADs), a sequence of the number of species that occur with particular frequency within a community [5], perhaps the most comprehensive presentation of species communities, are also affected. The SAD encompasses richness as its special case. Accordingly, a number of theoretical studies on SADs have been undertaken including those investigating the effect of sampling (e.g. [6–10]). These theoretical developments are based on how a sample SAD is shaped, when sampling is involved, from assumed species abundances, taking a hierarchical (or marginal) approach. Their common formulation relies on the fact that the sample SAD (after sampling) is described as a compound form of those, a sampling formula and an assumed distribution of abundances among species within a community. It is, however, unclear how sampling affects and changes the shape of original SADs (before being sampled) into resulting sample SADs, since the outcome relies upon assumptions made about the distribution of abundances among species. There has also been a variation in defining SADs among previous studies that conceals the distinction and induces an extra confusion.

Here, we take a conditional approach, a different approach from the previous studies, and articulate how the sampling bias is induced to the original SAD by random sampling. To do so, we describe the sampling issue using a formal theoretical setting, along with an illustrative example collected from an actual scientific marine survey [11–13] that was specifically designed for examining the effect of sub-sampling, and express the source of the issue by a deductive manner. Our focus is upon providing the reader with a comprehensive picture of the challenge of the sampling issue in ecological studies, rather than providing bias correction methods that tend to be survey specific and may restrict our perspective. This paper attempts to bring both theoretical and practical work together, reconciling the gap from a statistical perspective.

In the remainder of the paper, we describe the effect of sampling on the conditional modelling framework, without assuming any theoretical assumptions but that the original SAD is known, and illustrate the results empirically. This approach allows us to avoid extra assumptions which may affect the result in quantifying the effect of sampling. A description of the motivating data and the sub-sampling method employed is given in §3. It will also be shown in §4.1 that the (sub-)sampling framework can be described by a multivariate hypergeometric distribution in a simple random sampling context and it will also be reviewed in relation to the SAD (§4.2). Sections 5.1 and 5.2 quantify the effect of (sub-)sampling as attenuation of the original SAD. We show that the idea of rarefaction discussed in Hurlbert [2], Simberloff [3] and Heck *et al.* [4] is a special case of this attenuation. Both explicit and asymptotic forms of the attenuation are derived for the (sub-)sampling framework. These theoretical approaches are investigated using a re-sampling experiment using data collected by Heales *et al.* [12], and the discrepancy between the model and the data is also examined. Section 5 addresses some ecological perspectives in relation to the previous theoretical studies, and reconciles them on a statistical sampling framework. Section 6 gives some discussions about related topics followed by the concluding remarks (§8).

## 3. Data

### 3.1 The marine survey

The dataset used in this paper is one of the trawl bycatch samples from the west of Mornington Island in Australia's Northern Prawn Fishery in 1998 [12]. The purpose of the study was to determine the effect of sub-sampling on the estimate of species composition and abundance, particularly due to the samples being taken from different times on the conveyor of seawater hoppers. The catch chosen for this study was sub-sampled and all sub-samples were completely enumerated.

On the research vessel, large animals in the catch were first removed using an aluminium grid (300 mm) and a recirculating seawater hopper was then used to hold the catch before sorting. The entire catch in the hopper was extracted by the sorting conveyor belt and collected into consecutively numbered boxes (sub-sample replicates), each of which was about 10 kg. Each box number represented the chronological order of sub-sample extracted from the seawater hopper. The samples were identified to the lowest taxonomic level possible (mostly species). Where this was not possible, the data were grouped to genus and in a few cases to family. Total numbers and weights were recorded for each species in each sub-sample and entered directly into a relational database. See Heales *et al.* [12] for more details of the data collection method.

For this study, one trawl catch consisting of 116 species with 13  611 individuals was chosen. This was the largest catch over the trawls with a total catch weight of 334 kg. The entire catch was split into 26 boxes, each of which represents roughly 4% of the entire catch.

### 3.2 Sub-sampling experiment

To investigate the effect of taking different size sub-samples from the whole catch, the contents of several boxes were combined, repeatedly choosing boxes without replacement, and combining the data from each box into a single sub-sample. We varied the number of boxes chosen to generate a range of sub-sample sizes.

Let *x*_*kb*_ be the number of individuals of species *k* in box *b*, and whether species *k* is not observed in a subset of all 26 boxes $B_{i}\subseteq B=\{1,2,\ldots ,26\}$ is defined as $t_{ki}=\prod_{b\in B_{i}}I(x_{kb}=0)$. Each subset of boxes represents a sub-sample, indexed by *i* with *i*=1,2,…,*q*. Note here that *t*_*ki*_=1 means that species *k* is unobserved in all boxes in the set $B_{i}$, i.e. completely absent from the *i*th sub-sample. A set of boxes is randomly selected from the 26 boxes B without replacement, with the number chosen to approximate the required sub-sample size. The possible number of combination of boxes is equal to $26!/\{(26-|B_{i}|)!|B_{i}|!\}$ so we considered *q*=500 to be adequate for the experiments. Here, $|B_{i}|$ denotes the number of boxes in the set. However, when the number of boxes in the set $B_{i}$ is one, only 26 experiments are possible.

The empirical probability of species *k* being absent from the sub-sample, ${\hat{p}}_{k}$, is equal to

$${\hat{p}}_{k}=\frac{1}{q}\sum_{i=1}^{q}t_{ki}.$$

Note that 1/*q* is the unit resolution of ${\hat{p}}_{k}$ in this experiment. Each box contains a different number of individuals, so that the number of individuals in the *i*th sub-sample, *n*_*i*_, is also different. The sub-sampling ratio *r* is therefore calculated by

$$r=\frac{1}{q}\sum_{i=1}^{q}r_{i},$$

where $r_{i}=n_{i}/N=N^{-1}\sum_{b\in B_{i}}\sum_{k=1}^{K}x_{kb}$. Note that these calculations are based on counts of individuals, where *N* represents the number of individuals in the catch.

This combining-boxes experiment obviously assumes homogeneity over the sequence of specimens from the seawater hopper, and the model described in §4.1 also makes that assumption. This could be an issue when the assumption fails. However, it should always be expected that a kind of heterogeneity can be somehow unintentionally involved in the samples from field surveys. The detail will be investigated in §5.2 by looking at the discrepancy from the data.

## 4. The framework

### 4.1 Sampling in ecological studies

Given the data described earlier (§3), a sub-sample, the boxes combined, can be simply considered as an extracted part of the entire catch. We assume here that the population of our interest is the entire catch that consists of *N* individuals of *K* species which we sub-sample. Let ***X***=(*X*_1_,*X*_2_,…,*X*_*K*_) be the vector of random variables representing the number of individuals of each species observed in a sub-sample. The number of individuals, ***X***=***x***, acquired by a simple random sampling scheme without replacement, follows a multivariate hypergeometric distribution [14,15]:

4.1 Pr(X=(x1,x2,…,xK))=∏k=1KmkxkNn−1,

where *m*_*k*_ is the number of individuals of species *k* in the catch, $N=\sum_{k=1}^{K}m_{k}$, and the sub-sample size is $n=\sum_{k=1}^{K}x_{k}$. Note that the distribution here assumes that the probability of each individual being in a sub-sample is common regardless of their species or body size, *n*!(*N*−*n*)!/*N*!.

The procedure of sub-sampling simply deducts *x*_*k*_ individuals of species *k* from *m*_*k*_ according to the sub-sampling ratio *r*=*n*/*N*. The expected value of *X*_*k*_ is given as E[*X*_*k*_]=*rm*_*k*_. So it is likely that at least one individual of species *k* is observed in the sub-sample (*x*_*k*_≠0) if *m*_*k*_ is reasonably big with respect to *N*. This implies that sub-sampling may not affect inferences relating to species presence/absence and richness for abundant species. However, it is not immediately clear for rare species, that is, those that occur in relatively small numbers (abundances).

### 4.2 Species abundance distributions

The SADs are a useful representation of species composition defined as a sequence of the number of species that occur with particular frequency within a community [5]. Often SADs are defined informally in ecological literature, and there seems to have been a confusion referring to different distributions as the same SAD (see §6 for details). To avoid extra confusion, we here give a formal definition of SADs following the description by McGill *et al.* [5], and adopt it elsewhere in the paper. We consider the SAD of the catch ***m***=(*m*_1_,*m*_2_,…,*m*_*K*_) and a sub-sample ***X***=(*X*_1_,*X*_2_,…,*X*_*K*_). The mass of a particular frequency *j* of SADs for the catch and sub-sample are, respectively, defined as

4.2 $$\left.\begin{array}{rl} y_{j} & =\sum_{k=1}^{K}I(m_{k}=j)\\ \mathrm{and}\;Z_{j} & =\sum_{k=1}^{K}I(X_{k}=j) \end{array}\right\}$$

for $j\in N_{0}=\{0,1,2,\ldots \}$, where *I*(⋅) is an indicator function. The vectors ***y***=(*y*_0_,*y*_1_,…,*y*_*N*_) and ***Z***=(*Z*_0_,*Z*_1_,…,*Z*_*n*_) then represent the SAD of ***m*** and ***X***; we refer to ***y*** as the original SAD and ***Z*** as the sample SAD. There are obvious constraints such as $\sum_{j=0}^{n}y_{j}=\sum_{j=0}^{n}Z_{j}=K$, the number of species, and $\sum_{j=0}^{n}jy_{j}=N$ and $\sum_{j=0}^{n}jZ_{j}=n$, the number of individuals in the catch and sub-sample, respectively. Note that it is always true that the number of individuals in the sub-sample is always less than in the catch, i.e. *x*_*k*_≤*m*_*k*_, but the number of species that occur with a particular frequency can be greater in the sub-sample, *z*_*j*_, than in the catch, *y*_*j*_. It is, for example, always true that *y*_0_=0 but *z*_0_≥0; the sample SAD, ***z***, can include zeros, and is typically right skewed.

As defined above, SADs are exactly the same as what is called size index [16] or frequency of frequencies [7,17] in statistics. Note that the term ‘distributions’ used for SADs here can be a little misleading, and does not mean the distributions in the statistical sense, according to our definition of the SAD. It is not a probability distribution that governs the random variable *Z*_*j*_ but a sequence of (random) variables representing the number of species that occur with particular frequency (see §6).

## 5. The effect of sampling

### 5.1 Attenuation of species abundance distributions by sampling

The effect of sub-sampling is investigated as the discrepancy between the SADs of the total catch ***y*** and sub-sample ***z***. Given the total number of individuals in the catch *N* of species composition ***m*** (or ***y***) and the number of individuals in the sub-sample of size *n*, to determine the extent to which sub-sampling may reduce the original SAD, ***y***, the expected value of the sample SAD, E[***Z***], is needed. From the definition of ***Z*** (equation (4.2)), the expected value is derived as

5.1 $$\begin{array}{rl} E[Z_{s}] & =\sum_{k=1}^{K}Pr(X_{k}=s) \end{array}$$

5.2 =∑k=1KmksN−mkn−sNn−1

5.3 =∑j=1nyjjsN−jn−sNn−1

5.4 =∑j=1nyjnsN−nj−sNj−1=∑j=1nyjjs(n)s(N−n)j−s(N)j.

The symbol here (⋅)_*k*_ denotes the descending factorial moment that is defined as (*d*)_*k*_=*d*(*d*−1)⋯(*d*−*k*+1) with (*d*)_0_=1 for 0≤*k*≤*d*. The hypergeometric distribution in equation (5.2) stems from the multivariate hypergeometric distribution (equation (4.1)) as its marginal distribution. In ecological literature, Dewdney [8] is one of the earliest researchers to have investigated the sampling effect on SADs, and has reached an equivalent result as equation (5.3). Note that E[*Z*_*s*_] can be considered as a weighted total of the original SAD, *y*_*j*_ (equation (5.4)). The coefficient part or weight represents the attenuation factor of a biological quantity. From the definition, its variance is

5.5 $$\mathrm{Var}[Z_{s}]=\sum_{k=1}^{K}Pr(X_{k}=s)\{1-Pr(X_{k}=s)\}+2\sum_{k<l}\{Pr(X_{k}=s,X_{l}=s)-Pr(X_{k}=s)Pr(X_{l}=s)\}.$$

In a more general statistical context, Sibuya [18] also derived the same result in the study of statistical disclosure control, discussing the risk of revealing a singleton identifiable when data are publicly available.

It is useful to consider their asymptotic behaviour, that is, when the total number of individuals, *N*, is relatively large. This is not unreasonable as sub-sampling is commonly taken when catches are large. The asymptotic description of equation (5.4) simplifies to

5.6 E[Zs]≃∑j=1n∗yjjsrs(1−r)j−s,nN→r,N→∞,

where $n^{*}=\max\{j:y_{j}>0\}=\max_{k}\{m_{k}\}$ and *r* is the sub-sampling ratio. This result occurs since

$$\frac{{\left(n\right)}_{s}{\left(N-n\right)}_{j-s}}{{\left(N\right)}_{j}}=r^{s}{\left(1-r\right)}^{j-s}\exp\left(\frac{c(j,s,r)}{2N}+O(N^{-2})\right),\;\;(N\to \infty )$$

with a constant, *c*(*j*,*s*,*r*)=*j*(*j*−1)−*s*(*s*−1)*r*^−1^−(*j*−*s*)(*j*−*s*−1)(1−*r*)^−1^. Its variance (equation (5.5)) also simplifies to

5.7 Var[Zs]≃E[Zs]−∑j=1n∗yjjsrs(1−r)j−s2,nN→r,N→∞.

Both asymptotic forms involve the binomial form which is mathematically more tractable.

**Figure 1.**
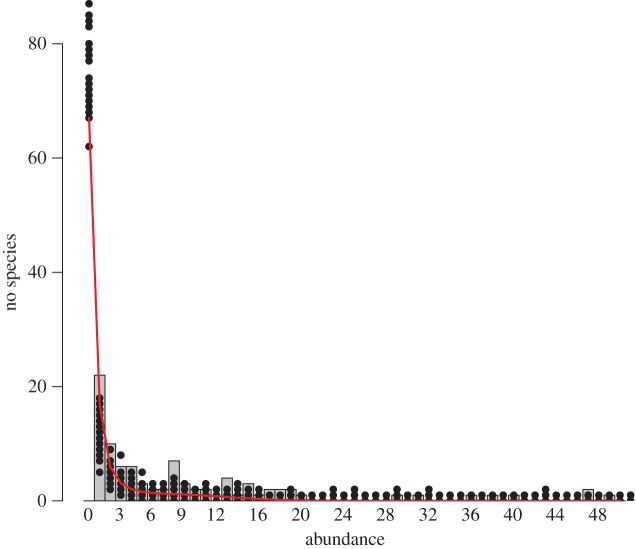
Species abundance distributions in the entire catch (bar plot), *y*_*j*_, and in the 26 sub-samples (dots), *z*_*j*_. The expected species abundance distribution of sub-samples with *n*=524 is superposed (red line), E[*Z*_*j*_].

The theoretical result (equation (5.4)) is investigated by considering the dataset. Figure 1 shows the appearance of three types of SADs: in the catch, the original SAD, *y*_*j*_, (bar plot), in each one of 26 boxes as a sub-sample, the observed sample SAD, *z*_*j*_, (dots) and the expected sample SAD, E[***Z***], (red line). The *x*-axis is restricted to abundances of less than 50 individuals. The mean number of individuals in the sub-samples was calculated as described in §3.2 and substituted into equation (5.4) with *n*=524. The theoretical line agrees well with the estimates from the sub-sampled data.

### 5.2 Risk of species absence in the sub-sample

In figure 1, there is obviously no species with zero abundance in the catch since only species observed in the catch were reported. Nevertheless, after taking sub-samples, a substantial number (approx. 70–80) of the species were absent, demonstrating the risk of information loss on those species due to sub-sampling. We now quantify this reduction by considering the case when a species is absent from a sub-sample. When *s*=0 in equation (5.4), the expected number of missing species in a sub-sample is

5.8 $$E[Z_{0}]=\sum_{k=1}^{K}Pr(X_{k}=0)=\sum_{j=1}^{n}y_{j}\frac{{\left(N-n\right)}_{j}}{{\left(N\right)}_{j}},$$

and its asymptotic form is given as

5.9 $$E[Z_{0}]≃\sum_{j=1}^{n^{*}}y_{j}{\left(1-r\right)}^{j},\;\left(\frac{n}{N}\to r,N\to \infty \right).$$

Equation (5.9) therefore represents the risk of a species being absent in the sub-sample.

**Figure 2.**
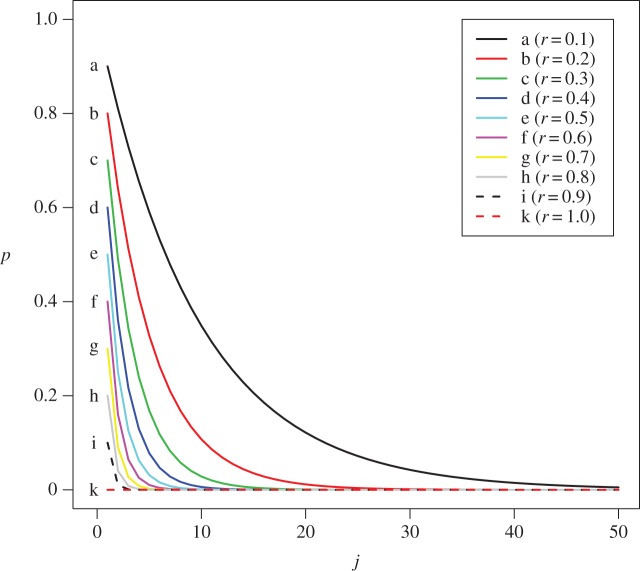
Traces of the attenuation factor *p*(*r*,*j*)=(1−*r*)^*j*^ for various values of sub-sampling ratio *r*. Each trace represent the theoretical probability of species absence in a sub-sample for different sub-sampling ratio.

The attenuation factor can now be described as a function of sub-sampling ratio *r* and species abundance of count *j*,

$$p(r,j)={\left(1-r\right)}^{j},$$

where (1−*r*) is commonly referred to in survey literature as the finite population correction term. When the entire catch is sorted *r*=1, and there is no information loss as E[*Z*_0_]=0. An intuitive interpretation of this asymptotic formulation is the risk where all *j* individuals of the species may not be present in a sub-sample, as (1−*r*) simply represents the probability that an individual is not in the sub-sample. The attenuation factor also indicates that the effect of sub-sampling is not equal over all species but dependent on each species' abundance, *j*. For those species with more than 20 individuals present, the risk of their absence in the sub-sample is relatively low and almost zero even if the sub-sampling ratio was only 20% (figure 2). For rare species, however, the risk of being absent from a sub-sample is high, even for large sub-samples.

Heck *et al.* [4] also pointed that E[*Z*_0_] can be represented by a geometric distribution and derived it by substituting *s*=0 in equation (5.2). Clearly, rarefaction curves *K*−E[*Z*_0_] are a special case of the attenuation of SADs.

**Figure 3.**
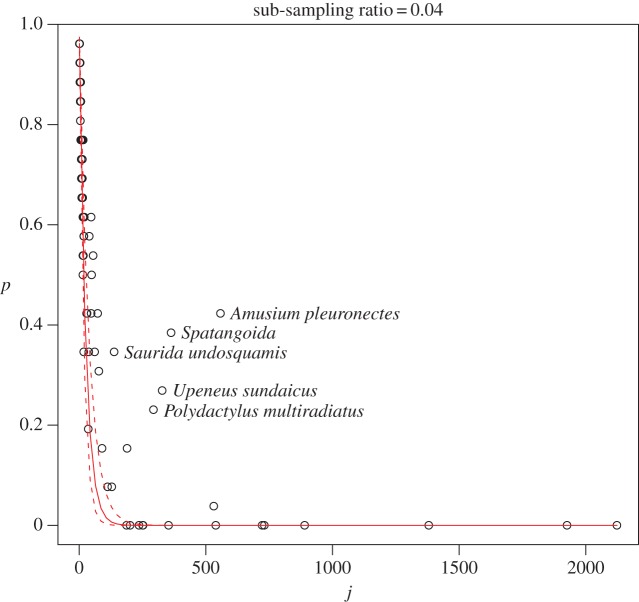
The calculated empirical probability of species absence in the sub-sample ${\hat{p}}_{k}$ and the theoretical line *p*=(1−*r*)^*j*^,*r*=0.04 (red solid line). The dashed lines are for the cases $\max\{r_{i}\}$ and $\min\{r_{i}\}$. The outlier species are labelled and they are potentially sampled non-randomly under the adopted sub-sampling procedure.

The performance of the attenuation factor *p*(*r*,*j*) is investigated using the dataset described in §3. In figure 3, the empirical probability of the *k*th species absence ${\hat{p}}_{k}$ is plotted against species abundance *j* for the catch when the sub-sample consists of only one box (circles). The superposed red line represents the attenuation factor with *r*=0.04 and the dashed lines are for the cases $\max\{r_{i}\}$ and $\min\{r_{i}\}$, where *i*=1,2,…,26 is a sub-sample replicate. The points are surprisingly well aligned with the theoretical line except for some outlier species that are labelled in figure 3. On close inspection of the data we discovered that these species were observed in particular boxes, which suggests it was a non-random sampling procedure for those species. According to the description of data collection procedure, these species would have been clustered in a particular chronological position on the sorting conveyor belt as Heales *et al.* [12] discussed. In fact, they identified Amusium pleuronectes (saucer scallop) as such a species.

**Figure 4.**
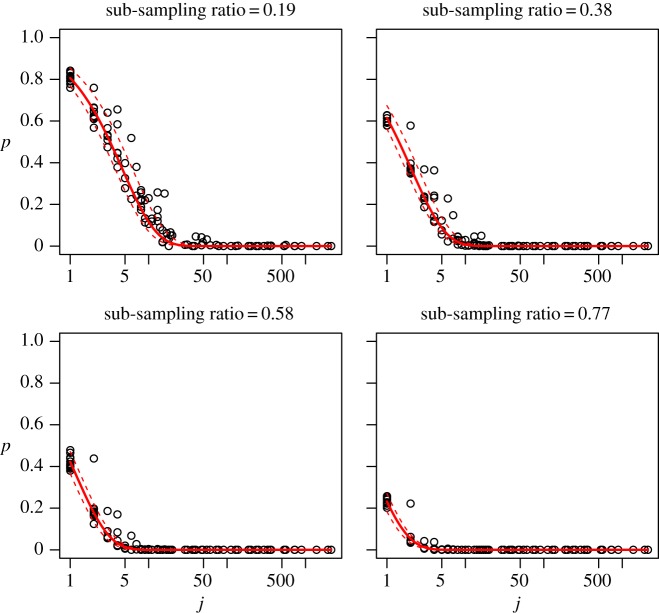
The calculated empirical probability of species absence in the sub-sample ${\hat{p}}_{k}$ and the theoretical line *p*=(1−*r*)^*j*^ (red solid line) and the cases $\max\{r_{i}\}$ and $\min\{r_{i}\}$ (dashed lines). For this case *q*=500 is chosen. The *x*-axis is represented in the natural logarithmic scale.

Similar results for the sub-sampling experiment involving larger sub-samples, namely for *r*=0.19,0.39,0.58 and 0.77, are displayed in figure 4. Note that the scale of the *x*-axis is shown as a log scale for illustrative purposes, which is different to that in figure 3. For the larger sub-samples as well, the theoretical line represents the data remarkably well.

## 6. Relationship with previous studies

Previous studies on SADs have investigated the sampling effect by examining, explicitly or inexplicitly, the expected value of the sample SAD, E[***Z***]. A key aspect here is that the expected sample SAD of a particular abundance (frequency) *s*, E[*Z*_*s*_], is, as we have shown, given as the sum of each species' probability at which the particular abundance *s* is observed. Recalling equation (5.1), this can also be described by a different approach from ours, in a hierarchical manner adopted by the previous studies [6–10] as

6.1 $$\begin{array}{rl} E[Z_{s}] & =\sum_{k=1}^{K}Pr(X_{k}=s)\\ & =K\int_{0}^{\infty }\sum_{m}f(s|m)f(m|\text{λ})f(\text{λ})\;d\text{λ} \end{array}$$

6.2 $$\begin{array}{r} =K\int_{0}^{\infty }f(s|\text{λ})f(\text{λ})\;d\text{λ}. \end{array}$$

In equation (6.1), the first term *f*(*s*|*m*), insert sampling formula, defines the sampling process by which the observed abundance *s* is deducted from the true abundance *m*, the second term *f*(*m*|λ) describes the extent to which the true abundance *m* varies by chance, and the third term *f*(λ) determines the variation of abundances among species. By introducing the hierarchical description above, the species abundance *m* is now regarded as random, although we have instead assumed it to be non-random (known) in our framework (§4.2). Note that the difference among species, *k* (*k*=1,2,…,*K*), in equation (6.1) has vanished as the parameter λ now governs the variation of abundances among species. As in a pioneer work by Fisher *et al.* [6], if *f*(λ) is chosen to be a gamma distribution and *f*(*s*|λ) to be a Poisson distribution, the integration of these becomes a negative binomial distribution, and its zero-truncation of the resulting formula leads to Fisher's log series. If a lognormal distribution is chosen for *f*(λ), it leads to the discrete lognormal (or Poisson–lognormal) by Preston [19], as Pielou [7] notes.

A key part of accounting for the sampling effect is the first two terms taking the sum with respect to abundance *m* in equation (6.1). A typical choice for it may be a compound distribution of binomial and Poisson distributions, assuming simple random sampling, which becomes

f(s|λ)=∑m=0∞p(s|m)p(m|λ)=∑m=0∞msrs(1−r)m−sλmm!e−λ=(rλ)ss!e−rλ,

where *r* is the sampling ratio as the sampling effect. This has been studied among researchers (see e.g. Dewdney [8], Green & Plotkin [9]). Green & Plotking [9] have also suggested a form of negative binomial distributions for *f*(*s*|λ) as an alternative, reflecting the idea that the regional sampling effect can be proportional to the mean abundance of the area under study, like the Poisson case above, *r*E[*M*]. We, however, note that the sampling effect can be proportional to the mean abundance. This is a special feature of the Poisson distribution, which does not hold for the negative binomial distribution under random sampling settings.

Instead of using equation (6.1), Dewdney [8] used equation (5.3) as we did but proposed a Poisson approximation to the hypergeometric term. This is misleading, since such approximation works when *N* and *j* or *n* tend to infinity and *jn*/*N* remains equal to λ. Note that both cases rescale *j* as λ but the original SAD, *y*_*j*_, is also defined on the original scale *j*. His conclusion has consequently slipped to equation (6.2) that subsequently confuses ***y***_*j*_ and *f*(λ) unfortunately, although they are not the same. We emphasize here that λ in equation (6.2) is the average abundance of species so the distribution *f*(λ) is not the same original SAD, *y*_*j*_, defined by equation (4.2), the number of species that occur with particular frequency within a community, whereas *f*(λ) defines the distribution of average abundance among species.

In fact, there are variations in ways of describing the SAD among previous studies. Some researchers refer to *f*(λ) as the SAD [9,10,20]. The normalized expected value of sample SADs, E[***Z***]/*K*, is also called relative SADs (e.g. [21]). They look like a probability function as its total mass is one, but we stress here that neither of these is the probability distribution that the number of species that occur with frequency *s*, *Z*_*s*_, follows.

The discussion above distinguishes our approach from the previous studies that mainly describe how the sample SAD is shaped from individual species abundances defined by *f*(λ). By contrast, our conditional approach, assuming the original SAD to be known, has shown the mechanism of how the original SAD, ***y***, changes its shape to the sample SAD, E[***Z***], owing to sampling. As shown (equations (5.4) and (5.6)), this is described as a weighted linear combination of the original SAD, *y*_*j*_, and the attenuation factor, *p*(*s*,*j*), as

6.3 $$E[Z_{s}]=\sum_{k=1}^{K}Pr(X_{k}=s|m_{k})=\sum_{j=1}^{n}p(s,j)y_{j},$$

where *j* is species abundance (the number of individuals). Equations (6.2) and (6.3) both provide the (expected) sample SAD, although their interpretations are very different. Nevertheless, this fact appears to have been overlooked and perhaps confused in the previous studies.

## 7. Discussion

### 7.1 Asymptotic properties of the attenuation factor

**Figure 5.**
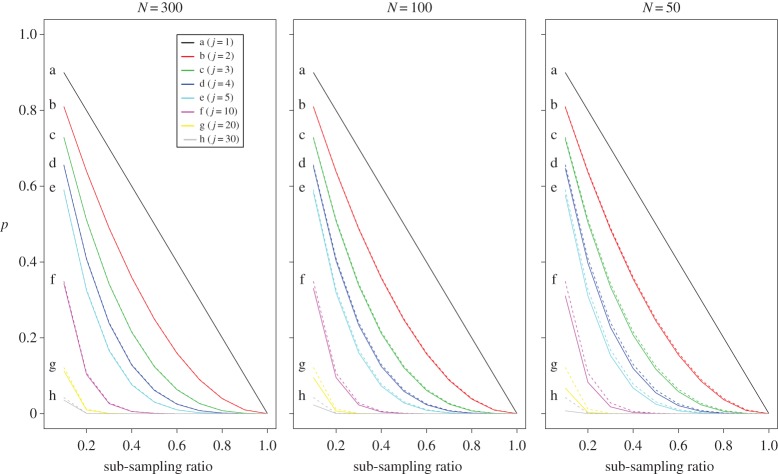
Comparison between exact (dashed line) and asymptotic (solid line) calculation of attenuation factor for each species abundance *j*.

Since the attenuation factor is asymptotically derived from equation (5.8), it is important to investigate appropriate conditions for use in actual situations. Figure 5 shows its asymptotic behaviour for different sizes of the catch, *N*. Note that for the case *j*=1, E[*Z*_0_] is not an approximation but an exact result (equation (5.8)). The approximation error tends not to be ignorable for species with relatively large abundance *j* when *N* is relatively small. However, as *j* increases, the size of attenuation factor decreases. By contrast, for large *N*, the approximation error tends to be quite small. In fact, little difference can be observed when *N*=300 (figure 5). Given that sub-sampling of the catch is common when *N* is large, this asymptotic approximation may work reasonably well in general situations.

### 7.2 Sub-sampling ratio based on weight

Up to this section, the sub-sampling ratio has been based on count of individuals, but this requires counting the number of individuals *N* of the entire catch; therefore, the sub-sampling ratio is often calculated using weights in marine surveys. For this situation, the sub-sampling ratio based on weight *r*^*w*^ can be expressed replacing *r*_*i*_ by

$$r_{i}^{w}=\frac{w_{i}}{W}=\frac{1}{W}\sum_{b\in B_{i}}\sum_{k=1}^{K}{\bar{w}}_{k}x_{kb},$$

where *W* is the total weight of the catch and ${\bar{w}}_{k}$ is mean individual weight of species *k*. If *r*≈*r*^*w*^, the sub-sampling ratio based on weight *r*^*w*^ is a substitute for *r*. It may be difficult to address a general condition where this assumption is satisfied. However, the sub-sampling experiment undertaken in this paper shows that the calculated sub-sampling ratio based on volume (number of boxes), *r*^*w*^=0.0385, was very close to that based on count, *r*=0.04, making it feasible to use *r*^*w*^ as an approximation of *r*. Using one dataset as an example in this study, it would be too ambitious to say that we can always expect this kind of agreement.

This implies another challenge of sampling issues, calculating an adequate value of the (sub-)sampling ratio, *r*, in field surveys. The nature of sampling stands on the fact that sampling is done on individual basis which may not follow the actual sampling protocol in surveys. Sampling can sometimes be undertaken instead by arbitrary sampling unit, such as weight, boxes and quadrants for example. This may cause a divergence in calculating *r* when heterogeneity is involved among the sampling units; see Gotelli & Colwell [22] for a comprehensive review of the distinction between individual- and sample-based data. This is not the limitation of the framework discussed here but an important strategy that needs to be considered before taking surveys, the sampling design and the way of analysing the data collected by the design.

### 7.3 Modelling over-dispersion

Our results show that any prediction of biodiversity such as species presence/absence and richness may need to be qualified if inferences from sub-sampled data are based on the common approach of normalizing the biological response by sub-sampling ratio. In fact, the prediction from sub-sampled data will underestimate species presence/absence and richness. For species presence/absence, it will be discounted by (1−*r*)^*j*^. For species richness, sub-sampling causes a bias E[*Z*_0_], so inferences made by using sub-samples tend to estimate fewer species than the actual richness *K*. As a consequence, the prediction model will be over-dispersed even if the assumed model was correct. The impact upon rare species can be more severe in the resource management context as there is a high risk of missing rare species completely.

## 8. Concluding remarks

The effect of (sub-)sampling on SADs, the extent to which the shape of original SADs is altered by sampling, has been quantified. The framework presented stands on simple random sampling of individuals from a finite population. The key to understanding the sampling effect has been identified as the attenuation factor, presented in both explicit and asymptotic forms. Reasonable agreement between the theory and data supports the potential remark of our theoretical result.

Throughout this paper, the results have been investigated and illustrated with the example data from a marine survey, but the proposed framework is not limited to marine applications, as among sampling taken in ecological studies there is a commonality: individuals (or equivalent) are, no matter what the sampling unit is, always the sampling target, that forms the sampling framework. However, the way of calculating (sub-)sampling ratio can be a slightly different story, and may depend upon its sampling unit, as heterogeneity among the sampling units, a divergence from the assumption of random sampling, can be involved.

The attenuation factor assumes that the sub-sample is taken randomly, a standard requirement of sampling design. This aspect may play a key role in some extent of further research directions. The performance or bias of (sub-)sampling procedures can be evaluated as the unexpected departure from the size of attenuation factor. The attenuation factor can be regarded as the benchmark of any (sub-) sampling process.

Taken together, our conditional approach has addressed the sampling effect on SADs as an attenuation factor, a function of sub-sampling ratio *r* and species abundance *j*. The sampling effect is, importantly, shown to be uneven among species, largely dependent on their abundance (number of individuals).

## Data accessibility

The dataset used in this study can be found in the electronic supplementary material.
